# Cost-effective analysis of childhood malaria vaccination in endemic hotspots of Bangladesh

**DOI:** 10.1371/journal.pone.0233902

**Published:** 2020-05-29

**Authors:** Abdur Razzaque Sarker, Marufa Sultana

**Affiliations:** 1 Population Studies Division, Bangladesh Institute of Development Studies (BIDS), Dhaka, Bangladesh; 2 International Centre for Diarrhoeal Disease Research, Bangladesh (icddr,b), Dhaka, Bangladesh; 3 School of Health and Social Development, Deakin University, Burwood, Melbourne, Australia; Guru Angad Dev Veterinary and Animal Sciences University, INDIA

## Abstract

**Introduction:**

Bangladesh has a history of endemic malaria transmission, with 17.5 million people at risk. The objective of this study was to assess the cost-effectiveness of universal childhood malaria vaccination in Chittagong Hill Tracts (CHT) of Bangladesh with newly developed RTS,S/AS01 malaria vaccines.

**Methods:**

A decision model was been developed using Microsoft® Excel to examine the potential impact of future vaccination in Bangladesh. We estimated the economic and health burden due to malaria and the cost-effectiveness of malaria vaccination from the health system and societal perspective. The primary outcomes include the incremental cost per Disability-Adjusted Life Year (DALY) averted, incremental cost per case averted, and the incremental cost per death averted.

**Results:**

Introducing childhood malaria vaccination in CHT in Bangladesh for a single birth cohort could prevent approximately 500 malaria cases and at least 30 deaths from malaria during the first year of vaccination. The cost per DALY averted of introducing the malaria vaccine compared to status quo is US$ 2,629 and US$ 2,583 from the health system and societal perspective, respectively.

**Conclusions:**

Introduction of malaria vaccination in CHT region is estimated to be a cost-effective preventive intervention and would offer substantial future benefits particularly for young children vaccinated today. Policies should, thus, consider the operational advantages of targeting these populations, particularly in the CHT area, with the vaccine along with other malaria control initiatives.

## Background

Malaria, an infectious disease, is still prevalent throughout tropical and subtropical regions of the world. According to the World Health Organization (WHO), approximately 212 million new cases and 0.42 million estimated deaths occurred globally due to malaria in 2015 [1]. The latest Global Burden of Disease 2017 data showed that neglected tropical diseases including malaria are responsible for approximately 62,300 thousands of global disability adjusted life years (DALYs), of which malaria alone contributed 72% of the total DALYs [2]. Malaria is a type of vector-borne disease transmitted through the bites of Anopheles mosquitoes; a tiny fraction of infections are transmitted via transfusion or congenital transmission routes [3]. Almost all malaria deaths are caused by *Plasmodium falciparum* and most of these deaths occur in African children less than 5 years old [3].

Bangladesh has a history of endemic malaria transmission in 13 out of its 64 districts, and approximately 17.5 million people are at risk, although only 27,737 cases were reported in 2016 [4]. However, it has been assumed that unreported cases might have been as high as 250,000 each year, highlighting the true burden of malaria disease in Bangladesh [5]. The number of malaria cases in these areas fluctuates seasonally, and the highest malaria incidence occurs during the rainy season, from April to October each year in Bangladesh whereas *Plasmodium falciparum* is the main malaria parasite in Bangladesh [4,6]. Despite apparent declines in prevalence, it still remains as a significant public health problem and a leading cause of hospital admissions during the rainy season in Bangladesh [7]. Among all cases, approximately 80% belong to the 3 most malarial-prone districts (Bandarban, Khagrachari and Rangamati), which are collectively known as Chittagong Hill Tracts (CHT); it has the highest malarial incidence reported within the country [7]. A number of other relevant factors, such as proximity to forest, household density and culture, and elevation made the CHT regions more vulnerable for malaria cases and CHT has been declared as a “malaria prone endemic area” [8], and known as malaria hotspots in Bangladesh. Malaria affects all of the population irrespective of age, sex, and occupations, though children are the most vulnerable [9]. In terms of health and economic burden, the costs of treating malaria cases vary amongst different socio-economic strata and geographical locations. Furthermore, due to the absence of financial security, expenses for treating malaria cases came exclusively from out-of-pocket payments [10,11]. Although malaria diagnosis and treatment is free in Bangladesh, however, many malaria patients spent for treatment care, including transport costs, diagnostic costs, and antimalarials [4,12]. The treatment costs have been shown to be proportionately higher for poor households, and are catastrophic to poorer households and rural dwellers [11].

According to World Health Organization, RTS,S/AS01 is the most advanced malaria vaccine candidate, which is a complementary malaria control tool that could potentially be added to-and not replace-the core package of proven malaria preventive, diagnostic, and treatment measures [13]. The prevention of disease burden and death through vaccination is one of the most cost-effective and public health achievements in developing world [14–16]. However, the vaccine is not the single important issue. the efficacy or effectiveness of the vaccine, disease burden, and financial issues are the common concerns of vaccine introduction [17, 18]. A vaccine introduction decision-making study in Bangladesh demonstrated that the Government and other technical personnel would only be convinced if the information on the burden of disease comes from the real setting [19]. Value for money has become an increasingly necessary criterion for vaccine introduction and, therefore, the cost-effectiveness analysis could contribute to guide decision making about introduction of vaccines versus other health interventions [20]. There are various cost-effectiveness studies available on different infection preventive vaccines in Bangladesh [21,22] but none of them are pertinent to malaria. Cost-effectiveness analysis study is, thus, required to implement sustainable control programs and to assess the future needs for malaria elimination. To address the literature gap, our intention was to conduct a cost-effectiveness analysis of malaria vaccination in Bangladesh and to highlight to policy makers whether or not there is a potential need for malaria vaccination introduction as a part of the malaria control initiatives. As a consequence, the objective of this study was to assess the cost-effectiveness of universal childhood malaria vaccination in CHT regions in Bangladesh with the newly developed RTS,S/AS01 malaria vaccines. In the light of other malaria vaccination studies [23–26], this analysis is limited to young children aged 3 years or less, as immunity tends to increase with increasing age [27].

## Materials and methods

### Model

**Fig 1:**
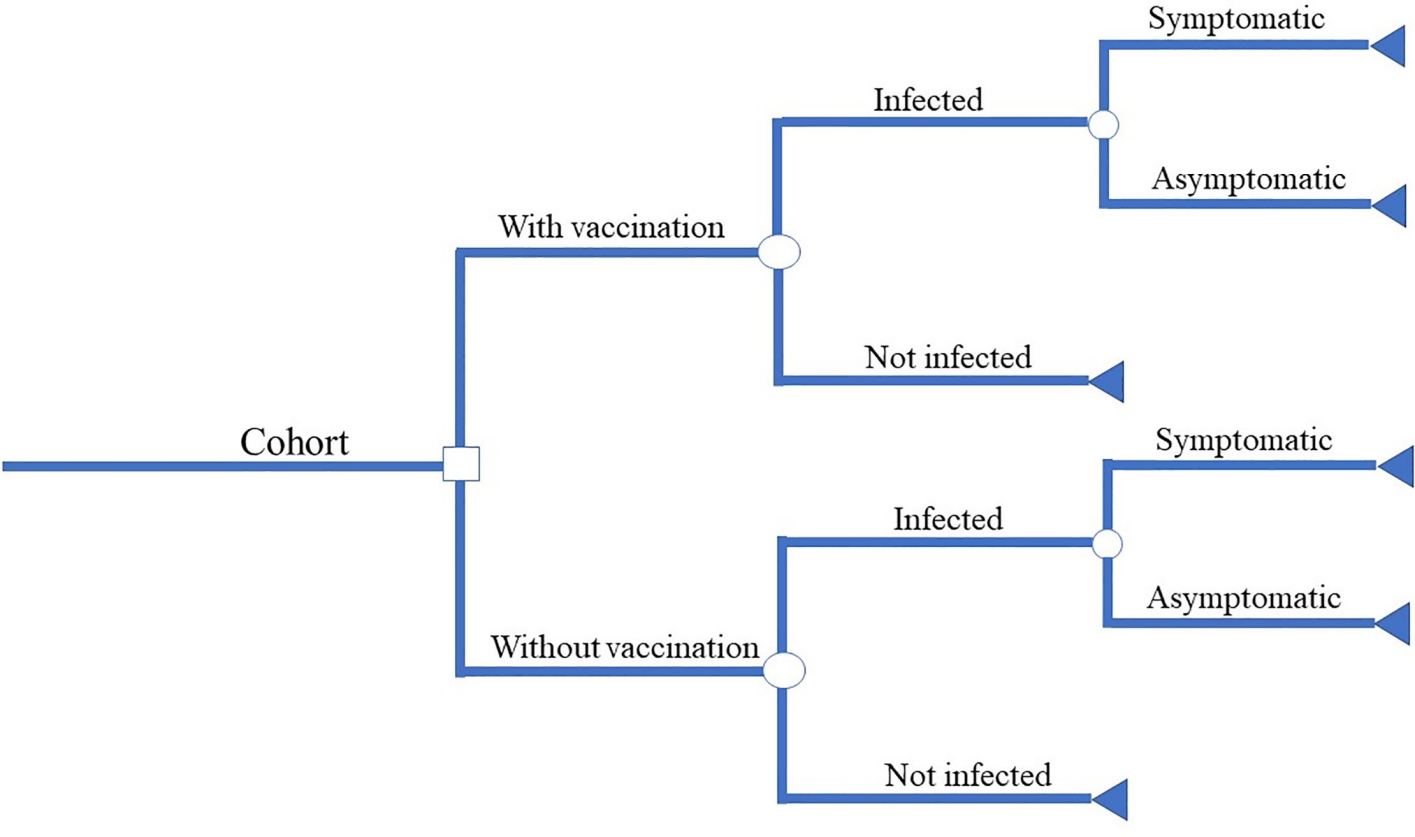
Decision tree for cost-effectiveness analysis of malaria vaccination.

A decision model was developed using Microsoft ® Excel to examine the potential impact of future vaccination in high risk, malaria-prone areas in Bangladesh. We estimated the economic and health burden due to malaria and the cost-effectiveness of malaria vaccination from the health system and societal perspective. The decision tree of cost-effectiveness analysis is shown in Fig 1.

The primary outcomes include the incremental cost per Disability-Adjusted Life Year (DALY) averted, incremental cost per case averted, and the incremental cost per death averted. Incremental cost-effectiveness ratios (ICERs) are calculated by dividing the cost difference with and without the universal childhood malaria vaccination program by the difference in health outcomes with and without the intervention. For these perspectives, several parameters were taken from various published papers and regional data sources. The various health associated outcomes, health care costs averted and the reduction of disease burden after the introduction of childhood malaria vaccines in regional settings were estimated in this model. Principal model parameters are described in Table 1. For comparing the pre- and post-malaria vaccination scenario, we estimated the potential events and possible costs to capture the baseline disease burden, and then assessed the number of malaria disease-associated events and possible costs that would occur after the introduction of the vaccination program. The model was applied to the 2016 annual static birth cohort (N = 146,255) based on national district web portal and the population projection of the Government of the People’s Republic of Bangladesh [28], and analysis was based on one year period. In addition, sensitivity analysis was carried out from 0.6 years up to 5 year time horizon [24,29,30]. The cost-effectiveness analysis was reported based on the health system and societal perspective according to the Panel on Cost-Effectiveness in Health in Medicine [31]. For the health system perspective, we included the medical care costs related to malarial episodes and the cost of vaccination program; whereas in the societal perspective, both direct medical (e.g., medicine, diagnostic), direct non-medical cost (e.g., transportation, lodging), and indirect cost (e.g., income loss) were included. However, the intangible costs, such as pain and discomfort were excluded from the estimation. In the current analysis, the costs averted by vaccination were subtracted from the costs invested in malaria vaccination program, divided by the DALYs or the number of deaths and cases averted. All future costs and benefits were discounted at a rate of 3% annually [32]. For reporting the cost-effectiveness scenario, we used the common cost-effectiveness threshold level proposed by the World Health Organization: an intervention is considered cost-effective if cost per DALY averted is less than three times of the national annual per capita GDP, whereas costs less than the GDP per capita are considered as highly cost-effective [33]. The concept of DALYs was used to quantify the disease burden incorporating life lost due to premature death and the time spent in unhealthy states [34]. The DALY is a time-based measure which combines years of life lost (YLL) due to premature mortality and the years of healthy life lost to living in a state of less than perfect health (years lost to disability or YLD) in a country-specific context [35]. Therefore, DALY is the summation of YLL+YLD, and 1 DALY can be considered as equivalent to one lost year of healthy life. Using DALYs, it is possible to measure the gap between current health status and an ideal situation, where everyone lives according to their life expectancy without disease and disability [36].The detailed understanding, theory, methodology, and limitations of the DALY estimation have been described elsewhere [34,37]. Like previous studies [38,39], the following four equations were applied to estimate the total DALYs avoided due to introduction of malaria vaccination in CHT regions. The four Eqs (1–4) are written as:

**Table 1:** Cost-effectiveness model parameters, with uncertainty ranges.

Input parameter	Value	Sensitivity
Fully vaccinate population	146,255 [28]	-
Vaccination campaign coverage (%)	65% [47]	40 [63] to 80% [49]
Overall protective effectiveness (%)	39% [23]	34.3 to 43.3 [23–25]
Duration of vaccine protection (years)	1 [29]	0.6 to 5 [Assumption]
Incidences (cases/1000)	4.3 [40]	2 to 11.34 [7]
Case Fatality Ratio (cases/1000)	0.23[5]	0.09–0.41[5]
Duration of illness (days)	07 [41]	3–10 [Assumption]
Daly weight	0.2 [41]	0.061 to 0.281[42]
Life Expectancy, (years)	70 [64]	65 to75 [Assumptions]
Outpatient cost for provider (US $)	5.48 [44]	2.26 to 23.65 [44]
Outpatient cost for households (US $)	16.64 [44]	9.14 to 37.99 [44]
Inpatient cost for provider (US $)	30.26 [44]	15.64 to137.87 [44]
Inpatient cost for households (US $)	32.24 [44]	26.99 to 288.79[44]
Vaccine purchasing cost per dose (US $)	0.16 [21]	0.1 to 5 [23,24,26]
Vaccine delivery cost per dose (US $)	0.835 [43]	0.5 to 1 [Assumption]
Vaccine recipient cost	0.02 [43]	0.01 to 0.1 [Assumption]
Discount rate (%)	3% [65,66]	1 to 10% [63]
GDP Threshold for DALYs *		
Very cost effective (per capita GDP**)	(US $) 1,466	
Cost effective (3* per capita GDP)	(US $) 4,398	

(*World Health Organization guideline

** Bangladesh Bank 2016)

$$DALY\;avoided_{t}=YLD\;avoided_{t}+YLL\;avoided_{t}$$ (1)

$$YLD\;avoided_{t}=\left\{\left[\left(1‐CFR\right)\times Eff_{t}\times Cover\times N\times I\right]\times Length\times DW\right\}$$ (2)

$$YLL\;avoided_{t}=\{\left[\left(CFR\times Eff_{t}\times Cover\times N\times I\right)/0.03\right]\times \left[1‐Exp\;\left(‐0.03\times LE\right)\right]$$ (3)

$$Total\;DALYs\;avoided_{t}=\sum_{t=0}^{Durr}\frac{{Dalys\;avoided}_{t}}{{\left(1+0.03\right)}^{t}}$$ (4)

In the above equations, Eff _t_ is the effectiveness of the malaria vaccine in year t, Cover is the percentage of young children that would be vaccinated if the vaccine were provided for free, CFR, I, and N are the case fatality rate, incidence of malaria, and number of young children, respectively; Length is average duration of illness (i.e., number of days sick with malaria), DW is the disability weight, LE is the life expectancy and Durr is the duration of the vaccine effectiveness. The incremental cost-effectiveness ratio (ICER) defined as the ratio of the change in costs of malaria vaccination program (compared to doing nothing) to the change in effects of the vaccination in terms cases, deaths and DALYs averted. The following equation is used for ICER:

Incremental cost-effectiveness ratio (ICER) = (C_1_ –C_0_) / (E_1_ –E_0_)

Where C_1_ and E_1_ are the cost and effect of the malaria vaccination program while C_0_ and E_0_ are the cost and effect of the comparator respectively. Principal model parameters are described in Table 1.

### Incidence and case fatality rate

Among the ten malaria-endemic countries of the WHO Southeast Asian region, Bangladesh is considered as hypo-endemic for malaria transmission, where 90% of malaria is caused by Plasmodium falciparum [40]. The prevalence of malaria is 0.92 per 1000 population in these endemic areas, however, children under five years of age were found to have a higher prevalence, i.e., 4.37 per 1000 young children [40]. The overall malaria related mortality was 0.09 per 1000 population which was higher for adult people (>14 years), followed by the younger group ages (4.1 to 14.0 years old), i.e., 0.40 vs 0.32 per 1000 population respectively [5].

### Duration of illness and DALY weights

The frequency of episodes of malaria and the characteristics of malaria disease varies depending on the infected individual’s age, genetics, type of parasite, and immune response from previous malaria infections, and the intensity and seasonality of malaria transmission [3]. Due to limitation of data, like earlier study, we assume that in moderate or high transmission areas, children commonly experience 4–6 febrile illnesses per year attributable to malaria [3]. Consistent with an earlier study, we assumed that without effective malarial treatment, the average duration of a malaria episode is 7 days [41]. We used the DALY weights as 0.20 to measure the pain, suffering, and discomfort associated with malaria diseases [41]. To observe the possible effects of the universal malaria vaccination, the sensitivity range goes from 0.12 to 0.281 [41,42].

### Malaria vaccination and cost

RTS, S/AS01 Plasmodium falciparum is a safe and moderately efficacious vaccine which is closer to domestic licensure and has already been implemented in endemic Africa [23–25]. With its safety and efficacy upheld in the phase 3 trial, the vaccine has received a positive regulatory assessment, however, further evaluation of RTS,S/AS01 is required for vaccine introduction at wider level in different countries [3]. On the light of earlier study, we assumed four doses schedules, first dose will be administered at six months, second and third before 9 months, and fourth dose at 27 months with overall vaccine efficacy will 39.0% (95% CI, 34.3–43.3) [23]. The cost-effectiveness of the vaccination was evaluated over 1 year time horizon with sensitivity analysis from 0.6 years up to 5 years [29,30]. As per vaccination study regarding infectious diseases, due to absence of data, we assumed that the Global Alliance for Vaccines and Immunisation (GAVI) will subsidize the malaria vaccine prices for Bangladesh, which will be approximately US 0.16 [21] and will vary from US$ 0.1 up to US$ 5.0, and additionally, vaccine delivery cost will be incurred accordingly [23–26]. Based on an earlier vaccination trial, it was assumed that an additional US$ 0.5 to US$ 1 will be incurred per person for delivery-related activities [43]. Furthermore, in case of societal perspective, additional vaccine recipient cost (e.g., travel cost, time cost) will be incurred.

### Cost of illness due to malaria infections

A systematic review of the published literature on the costs and cost-effectiveness of malaria interventions was carried our earlier [44], and the findings of the review revealed that the average outpatient cost per treating malaria was US$ 5.84 from health system perspective, while approximately US$ 22.48 was spent from societal perspective. For inpatients, i.e. those who had severe episodes of malaria, approximately US$ 30.26 was incurred from the perspective of health system while the societal average cost was estimated at US$ 64.50 [44]. Due to limited information, in light of the earlier study, we assumed that approximately 24% of the malarial cases sought treatment from hospital care and the remaining patients utilized the hospital outpatient services [45].

### Coverage of the vaccine

The Expanded Program on Immunization (EPI) having the highest priority in Bangladesh recommended that every child should complete the scheduled immunization within their first year of life [46]. Recently, a large oral cholera vaccine trial was conducted in urban Bangladesh where the migration rate is higher than in other parts of the country and found that the two dose vaccine coverage was at least 65% in that area [47]. In this analysis, we assumed that the malaria vaccination will have a moderate coverage. Indeed, the overall immunization coverage is about 86% in Bangladesh [48]. Similar to earlier studies, we used a vaccine coverage from 40% to 80% for uncertainty analysis in this model [49].

### Sensitivity analysis

One-way sensitivity analyses were conducted based on the published and unpublished values of each parameter, in order to ascertain the impact of uncertainty in input values on the cost-effectiveness ratio. In scenario analyses, the cost-effectiveness ratios were estimated using low or high values of selected parameters, and compared with the base-case scenario, i.e., no-vaccination strategy.

## Results

Input parameters, vaccination cost, health burden, and decision criteria of future universal malaria vaccination program are summarized in Table 1. Introducing childhood malaria vaccination in CHT in Bangladesh for a single birth cohort of 146,255 young children, is projected to prevent approximately 500 cases and at least 30 deaths from malaria during the 1st year of vaccination period. By implementing the vaccination program, a total of 206.64 Disability Adjusted Life Years (DALYs) could then be averted (Table 2). The results also demonstrated that approximately US$ 5,697 could be saved within the shorter time horizon whereas US$ 3,620 could be saved as a consequence of avoiding the inpatient visits from the health system perspective. Furthermore, considering the societal perspective, about US$ 15,858 in the form of indirect costs (e.g. time cost) and out-of-pocket cost (e.g. transportation) involved for seeking care could be saved by investing in the universal vaccination (Table 2).

**Table 2:** Key vaccination program outcomes over 1 year’s period.

Parameters	Health system perspective	Societal perspective
Number of vaccinations ('000)	146.26	146.26
Average costs per vaccine	0.16	0.16
Average delivery costs per fully vaccinated child	0.84	0.84
Total inpatients cost averted ('000)	3.62	7.48
Total outpatient cost averted ('000)	2.08	8.38
Total costs averted ('000)	5.70	15.86
Net discounted costs of the vaccination ('000)	543.31	533.73
Total number of cases averted ('000)	0.50	0.50
Total number of deaths averted ('000)	0.03	0.03
Total number of DALYs averted	206.64	206.64
Incremental cost- effectiveness ratio (ICER) per		
Case averted	1,089.84	1,070.62
Life saved	20,706.89	20,341.83
DALY averted	2,629.27	2,582.91
GDP Thresholds (for references)		
Cost-effective (3* GDP/capita)		US $ 1,466
Very cost-effective (GDP/capita)		US $ 4,398

(*World Health Organization guideline

** Bangladesh Bank 2016)

**Fig 2:**
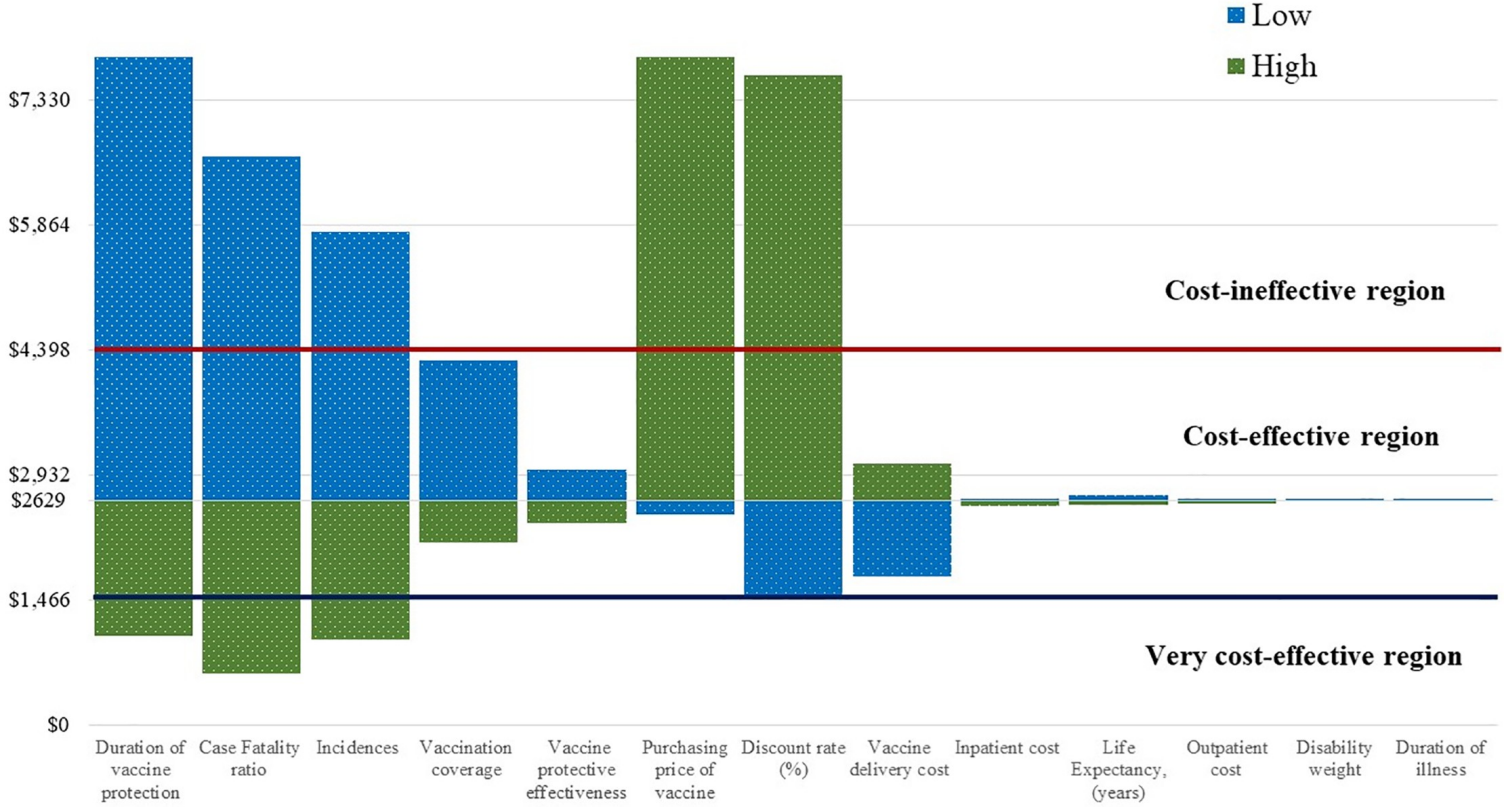
Cost per DALY averted: Health system perspective.

The cost-effectiveness estimates of this study demonstrated that the universal childhood malaria vaccination appeared to be a cost-effective investment both for health system and societal perspective, even with the lower efficacy of the current vaccine. By introducing the malaria vaccine compared to status quo, the cost per DALY averted is US$ 2,629 and US$ 2,583 from the health and societal perspective, respectively. Incremental cost-effectiveness ratios (ICERs) for both perspectives fell below the GDP per capita in Bangladesh (US$ 1,466) of 2015–2016 fiscal year, which was used as a threshold for determining the cost-effectiveness of any intervention. Therefore, these results demonstrate that cost-effectiveness of universal malaria vaccination program in CHT, Bangladesh laid between “very cost-effective” and “cost-ineffective region”, according to WHO criteria (Fig 2).

**Fig 3:**
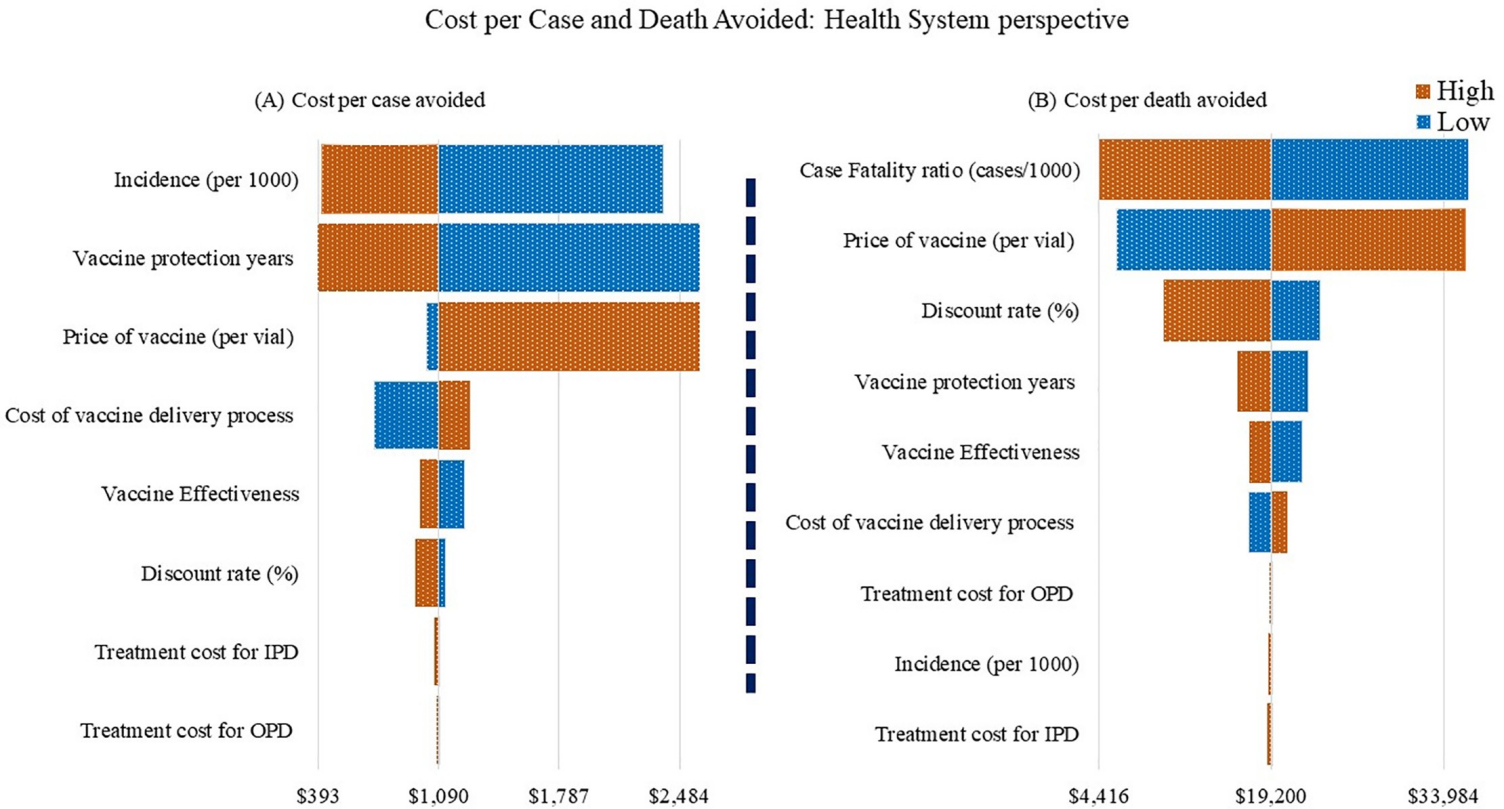
Cost per case and death averted: Health system perspective.

We assessed the robustness of the impact predictions by varying key model inputs (Table 1). Our scenario analysis found that the most influential parameters were duration of vaccine protection years, case fatality ratio, incidence, price of the vaccine, discount rate, vaccination coverage, and the cost of vaccine delivery-related activities from the health system perspective (Fig 2). The same parameters were also considered influential for societal perspective. However, vaccination becomes attractive if the longer time horizon is considered, and it is measured as a very cost-effective investment. Further, if the price would reduce to US$ 0.1, the vaccination program would be a very cost-effective investment, while at the highest price (US$ 5), it became a cost-ineffective option (Fig 2). Whilst the higher incidence and higher mortality rate of malaria will make the immunization program attractive and cost saving option from the health system perspective, a similar scenario is also observed from the societal perspective. Vaccine coverage and duration of protection years are other influential parameters for vaccine introduction into the CHT regions. Most of the above parameters (e.g. duration of vaccine protection years, case fatality ratio, incidence, and price of the vaccine) also appeared as influential parameters in this regard, considering cost per case and death averted due to vaccination (Fig 3).

## Discussion

Bangladesh has made significant progress towards malaria control, and from 2000 to 2014, malaria incidence has been reduced approximately 75% by introducing vector control interventions [50]. However, malaria is still a devastating health problem in endemic regions of the country, where approximately 156.6 million people are at risk of malaria infections [51]. Globally, there are various malaria control initiatives, such as insecticide-treated mosquito nets (ITNs) including long-lasting insecticidal nets (LLINs), indoor residual spraying (IRS), artemisinin combination therapy (ACT), seasonal malaria chemoprevention (SMS), rapid diagnostic tests (RDTs), intermittent preventive treatment (IPT), whose efficacy and effectiveness have been repeatedly demonstrated over many years [52–56]. Since malaria prevalence is highest in CHT region than in other endemic districts of the country, resources should be engaged in such a way that it could optimize the benefit of investment and reduce the malaria burden from this malaria hotspot area. Community participation is vital for controlling and preventing malaria disease, however, as a component of prevention strategies, malaria vaccination might be another alternative option, since benefits of vaccines are well known as they avert infections directly (immunization) and indirectly (via herd immunity) [15].

In Low- and Middle-Income Countries like Bangladesh, the introduction of new health technology such as new vaccines are expected to implement with delays. For instance, it takes years or even decades after the invention [57,58]. We have estimated the potential cost-effectiveness of malaria vaccination in terms of cost per life saved, death and DALYs averted which might be capable to guide upcoming malaria vaccine policy formulation and nationwide vaccine implementation. In a resource poor country like Bangladesh, precise information, when there are competing health priorities and other national and local priorities, is a prerequisite for policy makers [58]. Therefore, this study could be referenced as a starting point for such discussion. Our estimation demonstrated that introducing the universal childhood malaria vaccination in CHT region could avert approximately 500 cases and approximately 30 deaths during the first vaccination year, using direct protection of vaccines. The cost per case and death averted are US$ 1,090 and US$ 20,707, respectively, for health system perspective, which are slightly higher than societal perspective.

Again, the incremental cost per DALY averted is also within the ranges of cost-effectiveness analysis using WHO GDP criteria. Once the vaccination is fully implemented in the endemic districts, the vaccine effectiveness will be higher, incorporating distinct characteristics of herd immunity of infectious vaccines [39]. Compared to the other malaria prevention strategies, the effectiveness of RTS,S/AS01 is less attractive intervention than other preventive control measure even in African regions. A systematic literature review reported that the incremental cost-effectiveness ratio per DALY averted was $27 (range $8.15-$110) for ITNs, $ 143 (range $ 135-$ 150) for IRS, and $ 24 (range $ 1.08-$ 44.24) for IPT [44]. A mathematical model for an African setting predicted that by introducing RTS,S in every 100,000 fully vaccinated children, approximately 116,480 clinical cases and 484 deaths could be averted in a 15 year time horizon, where the cost per DALY averted was US$ 87 [59]. Another Sub-Saharan African study estimated the cost per DALY averted ranges between US$ 44-US$ 279 and considered as a ‘secondary intervention’ and not prioritized due to their high cost than WHO recommended other preventive interventions like SMS, IRS and even LLINs [26]. In a recent study, Galactionova and colleagues reported that RTS,S/AS01 could reduce malaria burden substantially in 43 endemic Sub-Saharan African regions and the median cost per DALY averted would be US$ 188, which is a highly cost-effective intervention in those contexts [24]. However, most of the cost-effectiveness studies have been conducted in African regions where the malaria prevalence is higher than in Asian region, and are, thus, not comparable, although the context-specific cost-effectiveness study is crucial for such decision making process [26].

In scenario analysis, we observed that incidence, case fatality rate, price of the vaccines, coverage, and protective effectiveness are the major influencing parameters in the analysis, which make the vaccination program attractive. Due to limited information about malaria related clinical data, we have adopted the parameter values from various sources which was reflected in the sensitivity analysis. Introduction of malaria vaccine in this context requires significant financial resources. For introduction of malaria vaccination program, price of the vaccine is critical. Although the price reported here was based on GAVI subsidization, additional vaccine-related costs will be incurred (i.e., cost of vaccine delivery). Therefore, in the future, locally produced vaccine would be highly recommended, which would reduce the price of the vaccines and make vaccination more financially affordable. RTS,S vaccination trial has not been administered yet in CHT region or in any endemic region of the country; therefore, the country’s real vaccine efficacy-related data are missing, however, such studies have been conducted in African regions [23–26,60]. Cost-effectiveness analysis presented in this study relies on generic assumptions of various parameters which will be different across regions, particularly where the prevalence is low and the labor cost is high.

Although this analysis concludes that the vaccination would be highly cost-effective investment in CHT region, however, a number of limitations should be taken into account as we made several assumptions which could affect the cost-effectiveness ratio. Since the malaria vaccine protection years figure is very low compared to other infectious vaccines; WHO does not yet recommend the inclusion of RTS,S/AS01 in the Expanded Programme of Immunisation due to this lower efficacy of vaccine [61]. Therefore, future development of vaccines considering the higher protective efficacy is prerequisite for such decision [61]. Again, due to unavailability of nationwide vaccine effectiveness and epidemiological data, we used various sources of data. Further, CHT had some distinct characteristics such as healthcare seeking behaviour, vaccine uptake, and lifestyle. Therefore, the actual scenario might be different than we found in our study. The country representative data (e.g., incidence rate, vaccination coverage) might be higher or lower, which can be handled via adequate sensitivity analysis. The other important limitation was that, we used static model rather than dynamic model. The dynamic model is most useful for predicting the cost-effectiveness of infectious disease control program as the indirect effect of reduced transmission is a significant public health gain. However, the dynamic model provided the higher cost-effectiveness ratio than the static model as higher coverage rate reduce the transmission of the disease, therefore, the result could be underestimating the actual benefit of the malaria vaccination program [59,62]. However, the advantage of static model is that it requires minimum data, can be built and understood easily and has a low cost. It was not possible to collect rigorous information that are essential for the dynamic model. It is often laborious to capture the seasonal factors even the probability that an unvaccinated susceptible is infected either from the environment or from direct contact due to presence of a single unvaccinated infective in such distinct area in CHT region. Although we did not compare the vaccination with other existing malaria control initiatives, RTS,S/AS01 has the potentiality for substantial reduction of malaria-related mortalities and morbidities, which suggests that malaria vaccination could potentially be a complementary intervention with other malaria control initiatives, especially in high malaria endemic districts of Bangladesh.

## Conclusions

Introduction of malaria vaccination in CHT region is estimated to be a cost-effective preventive intervention and would offer substantial future benefits particularly for young children vaccinated today. Policies should, thus, consider the operational advantages of targeting these populations, particularly in the CHT area, with the vaccine along with other malaria control initiatives. A malaria surveillance targeting endemic is also recommended for addressing the data related gap which would enable to implement an effective malaria control program in high endemic districts of Bangladesh.
